# Peptides with 6-Aminohexanoic Acid: Synthesis and Evaluation as Plasmin Inhibitors

**DOI:** 10.1007/s10989-016-9555-3

**Published:** 2016-09-19

**Authors:** Maciej Purwin, Agnieszka Markowska, Irena Bruzgo, Tomasz Rusak, Arkadiusz Surażyński, Urszula Jaworowska, Krystyna Midura-Nowaczek

**Affiliations:** 1 Department of Organic Chemistry, Medical University of Bialystok, Mickiewicza 2A Str, 15-222 Białystok, Poland; 2 Department of Physical Chemistry, Medical University of Bialystok, Mickiewicza 2A Str, 15-222 Białystok, Poland; 3 Department of Medicinal Chemistry, Medical University of Bialystok, Mickiewicza 2A Str, 15-222 Białystok, Poland

**Keywords:** 6-Aminohexanoic acid, Plasmin inhibitors, Antifibrinolytics

## Abstract

Fifteen new peptide derivatives of ɛ-aminocaproic acid (EACA) containing the known fragment –Ala–Phe–Lys– with an affinity for plasmin were synthesised in the present study. The synthesis was carried out a solid phase. The following compounds were synthesised: H–Phe–Lys–EACA–X, H–d-Ala–Phe–Lys–EACA–X, H–Ala–Phe–Lys–EACA–X, H–d-Ala–Phe–EACA–X and H–Ala–Phe–EACA–X, where X = OH, NH_2_ and NH–(CH_2_)_5_–NH_2_. All peptides, except for those containing the sequence H–Ala–Phe–EACA–X, displayed higher inhibitory activity against plasmin than EACA. The most active and selective inhibitor of plasmin was the compound H–d-Ala–Phe–Lys–EACA–NH_2_ which inhibited the amidolytic activity of plasmin (IC_50_ = 0.02 mM), with the antifibrinolytic activity weaker than EACA. The resulting peptides did not affect the viability of fibroblast cells, colon cancer cell line DLD-1, breast MCF-7 and MDA-MB-231 cell lines.

## Introduction

Plasmin is a serine protease which is involved in many physiological processes such as wound healing, tissue repair and migration in addition to its main role in fibrin cleavage. Plasmin inhibition is crucial in preventing plasmin over-activity, i.e. in blood coagulation disorders or during surgeries. Plasmin inhibitors have also been tested for other disease states including angiogenesis, cell proliferation, metastasis and embryo implantation.

Plasmin (PL) is distributed in the form of zymogen called plasminogen (PLG) Activation of PLG to PL is a result of a single cleavage of the scissile bond between Arg^561^ and Val^562^ in plasminogen (Robbins et al. 1967). PLG is produced as a protein which consists of 810 amino acids. During secretion a short 19 amino acid peptide is cleaved to produce a mature zymogen which consists of 791 amino acids. PLG is a single chain protein whose activation results in a two-chain disulfide-linked plasmin which is a serine protease with specificity similar to trypsin cleaving after Lys and Arg. The amino-terminal heavy chain of PL consists of five kringle domains, each containing 80 amino acid residues. The C-terminal light chain of plasmin is a typical serine protease (SP) which contains the catalytic triad composed of His^603^, Asp^646^ and Ser^741^.

**Fig. 1:**
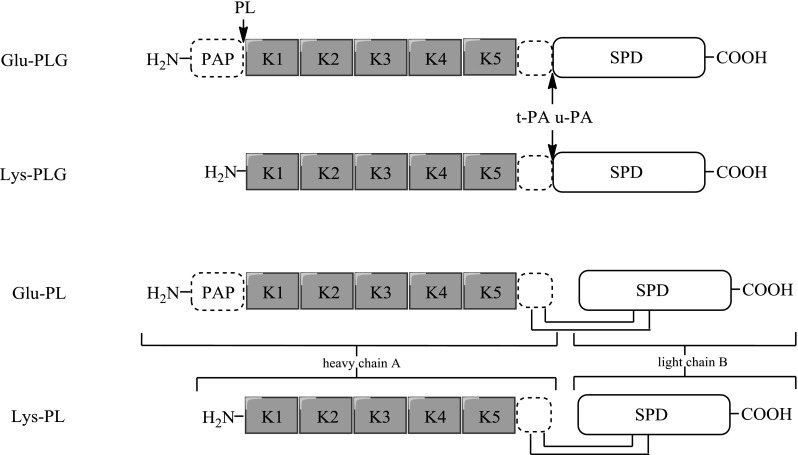
Forms of plasminogen and plasmin: one-chain Glu- and Lys-plasminogen and two-chain disulfide-linked Glu- and Lys-plasmin

Both plasmin and PLG exist in two forms which differ by sequences at the *N*-terminus (Fig. 1).

Glu-plasmin(ogen) (Glu-PL(G)) consists of glutamic acid at the N-terminus and possesses tight conformation (Cockell et al. 1998) while Lys-plasmin(ogen) (Lys-PL(G)) with lysine at the *N-*terminus has a much more relaxed structure (Castellino and Ploplis 2005). PLG is activated by tissue PLG activator (tPA) and, to a lesser degree, by urokinase PLG activator (uPA) or a complex of bacterial metabolites from *Streptococcus haemolyticus* and *Staphylococcus aureus.* The main role of t-PA is the dissolution of blood clots in vessels while u-PA is involved in mediating cell-related proteolysis. A number of studies have confirmed the role played by u-PA in embryogenesis (Berg and Menino 1992), embryo implantation and fertilisation (Sappino et al. 1989; Huarte et al. 1993), angiogenesis (Pepper et al. 1996), cancer and metastasis (Carroll and Binder 1999).

Plasminogen activation by tPA requires the presence of fibrin to which PLG and tPA bind via LBS located in the kringle domain of PLG (Lerch et al. 1980). Once formed, plasmin action is confined to the fibrin surface due to the kringle-mediated binding of plasmin to fibrin (Lucas et al. 1983). Plasmin proteolytically cleaves the fibrin clot and, as a result, restores blood flow to the affected tissues. The most important function of plasmin is intravascular thrombolysis although the proteolytic activity in normal and pathological conditions causes cell migration, inflammation and tissue remodelling (Carmeliet and Collen 1995). The evidence also suggests a less defined function of plasmin in a number of physiological and pathological processes relating to hormones, immunology, fertility, inflammation, bone formation, extracellular matrix degradation, cell migration and tissue remodelling. The effects of plasmin are specific, require the active catalytic centre and can be antagonised by lysine analogues, implying binding of the plasmin molecule to the cell membrane through its lysine binding sites.

**Fig. 2:**
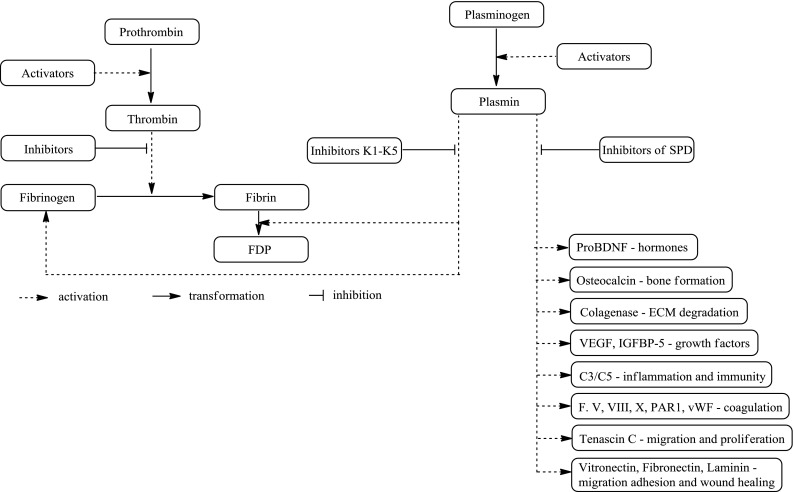
Scheme of blood coagulation and fibrinolysis with a wide role of plasmin

Inhibition of the fibrinolytic system is mediated by plasminogen activator inhibitors, mainly by PLG activator inhibitor PAI-1, and by plasmin inhibitors, mainly α_2_-antiplasmin, which exist in high concentrations in plasma and rapidly inactivate any free plasmin that could appear outside a blood clot (Moroi and Aoki 1976). Fibrinolysis plays a crucial role in blood clot degradation, cell invasion, but also embryogenesis, embryo implantation, ovulation and brain barrier function (Collen and Lijnen 1991; Vassalli et al. 1991) (Fig. 2).

At present, analogues of lysine, including ε-aminocaproic acid (EACA), are commonly used as fibrinolysis inhibitors. EACA is a synthetic derivative of the amino acid lysine which produces reversible blockade of lysine binding sites (LBS) of kringle 1 and 4 on PLG molecules, thus preventing PLG binding to fibrin and reducing the conversion of PLG to plasmin which is responsible for the degradation of blood clots (Kahar et al. 2009; Bhavanis et al. 2013). EACA and tranexamic acid (TXA) exert their effect by inhibiting the protein–protein interaction between PLG and fibrin. They work by reversible prevent the protein–protein interaction via the blockade of LBS in the kringle domain (Hochschwender and Laursen 1981). EACA and TXA are widely and effectively used as adjuvants to decrease surgical field bleeding, reduce blood loss and subsequent need for blood transfusion in oral, orthopedic, spinal, cardiac surgery, liver transplantation and prostate surgery (Choi et al. 2009; Ortmann et al. 2013).

Activation of PLG to plasmin occurs in tissues where the proteolytic cleavage of biological barriers is needed i.e. wound healing, inflammation, inflammatory diseases including atherosclerosis and arthritis, ovulation and trophoblast implantation, angiogenesis, nerve regeneration, and cell migration and proliferation during cancer. Active plasmin is formed close to the surface of tumor cells from PLG bound to cells. PLG is activated by the urokinase-type PLG activator which is produced by cancer or stroma cells. In physiological conditions, PL and uPA are inactivated by protease inhibitors such as α_2_-AP and PAI-1 present in the blood. Both uPA and plasmin degrade most of ECM components directly or through the activation of matrix metalloproteases (MMPs) (Kucharewicz et al. 2003; Kwaan and McMahon 2009). Plasmin is also responsible for the proteolytic activation of growth factors, including hepatocyte growth factor (Shanmukhappa et al. 2009), fibroblast growth factor (George et al. 2001) and transforming growth factor (Maeda et al. 2009).

Plasmin inhibitors are mostly classified as serine protease inhibitors. They bind targets through a highly conserved loop and form a reversible binding complex. A large number of compounds have been synthesised (Al-Horani and Desai 2014) but no drugs acting as short peptidyl plasmin inhibitors have been registered to date. The compounds did not meet drug registration requirements despite their increased activity. Consequently, the research for novel plasmin(ogen) binding inhibitors involves mimetics of lysine with modified functional groups (Fuji et al. 1972; Okada et al. 1988) or small peptides consisting of EACA (Westlund et al. 1982; Muramatu and Fuji 1971). Further research led to the synthesis of specific compounds such as H–d-Val–Leu–Lys–NHC_7_H_15_ (Fareed et al. 1981) which showed only the antifibrinolytic activities of plasmin, but sometimes slight activation was observed (Nagamatsu et al. 1963). The optimal specificity for plasmin inhibitors seems to be Phe–Lys (Bajusz et al. 1981). This cleavage sequence has been identified in many natural and synthetic substrates (Backes et al. 2000). Derivatives of short peptides with C-terminal lysine carboxyl groups transformed into aldehyde (Friberger et al. 1982), chloro- and fluoromethyl ketone (Angliker et al. 1988; Ganu and Shaw 1987) or *p*-nitroanilide (Collen et al. 1980) are active directed inhibitors or synthetic substrates of plasmin.

During our investigation we combined EACA with Lys (Midura-Nowaczek et al. 2003), S–Bzl–Cys (Midura-Nowaczek et al. 1994) or Nle (Midura-Nowaczek et al. 1996). These analogs showed variable antifibrinolytic activity relative to EACA (Bruzgo et al. 2006). The most selective inhibitor of the amidolytic activity of plasmin was Boc–EACA–Lys–EACA–NH_2_. Only the dipeptides Boc–Lys(Z)–EACA–NH_2_ and Boc–Lys(NH_2_)–EACA–NH_2_ appeared to be weak antifibrinolytics (Purwin et al. 2009).

The tripeptide H–d-Ala–Phe–Lys is known as a trigger in some antitumor prodrugs activable by plasmin De Groot et al. (1999, 2000, 2002). In our previous work we presented some antifibrinolytics such as heptyl amides and esters of tripeptides: Ala–Phe–Lys–NHC_7_H_15_ (Midura-Nowaczek et al. 1996, 2006). Similar compounds with this sequences were the acid and amides of (d or l)-Ala–Phe–Lys–OH(NH_2_) (Markowska et al. 2007). l-amino acid in the P3 position of tripeptides and their amides result in more effective inhibition than d-enantiomer. This result is in agreement with our previous research findings on the same tripeptide methyl ketones (Markowska et al. 2006). According to the literature, tripeptide synthetic substrates with d-configuration in P3 have better affinity for plasmin (Friberger et al. 1982).

Therefore, a new structure based on the tripeptide-spacer –Ala–PheLys- and EACA was designed and proposed as new serine protease plasmin inhibitors and/or antifibrinolytics. There are no high specificity and low-dose drugs in current clinical use which act as inhibitors of the active site of plasmin and kringle structures simultaneously. There are only two registered antifibrinolytic drugs which inhibit LBS in the kringle structures of plasmin: EACA and tranexamic acid. Aprotinin is the sole inhibitor of the active centre of plasmin in clinical use and is derived from bovine pancreas (single-chain 58 amino acids polypeptide). In view of the considerable overexpression of plasmin in many pathological states a search for biologically active compounds which inhibit the activity of this enzyme appears important.

Fifteen substituted peptides with the general formula: X–Phe–Lys–EACA–Y and X–Phe–EACA–Y where X = H, H–d-Ala, H–Ala and Y = OH, NH_2_, NH–(CH_2_)_5_–NH_2_ were synthesised using a solid phase and their effects on fibrinolytic and amidolytic activity were examined. The primary sequence of the peptides was –Ala–Phe–Lys–EACA– which was modified in order to establish structure–activity relationships. Their influence on other enzymes such as thrombin, tPA, uPA, kallikrein and trypsin were also tested. The compounds were tested in the cytotoxic test against breast cancer cell lines MCF-7 and MDA-MB-231 and against colon cancer cell lines DLD.

## Experimental

### Reagents

Rink amide resin, chloranil, acetaldehyde, HOBt = 1-hydroxybenzotriazole and TNBS = 2,4,6-trinitrobenzenesulfonic acid (1 % solution in DMF) were purchased from Fluka (Schnelldorf, Germany). 2-Chlorotrityl chloride resin, 1,5-diaminopentanetrityl resin, Fmoc–EACA–OH (Fmoc = 9-fluorenylmethyloxycarbonyl, EACA = 6-aminohexanoic acid), TFA = trifluoroacetic acid, DIPEA = diisopropylethylamine, piperidine, TBTU = tetrafluoroborate salt of the *O*-(benzotriazol-l-yl)-*N*,*N*,*N*′,*N*′-tetramethyluronium tetrafluoroborate, NMP = 1-methyl-2-pyrrolidon, Fmoc–Ala–OH, Fmoc-d-Ala–OH, Fmoc–Lys(Boc)–OH (Boc = benzyloxycarbonyl), Fmoc–Phe–OH were obtained from Iris Biotech GmbH (Marktrewitz, Germany). DCM = dichloromethane, DMF = dimethylformamide and methanol were the products of Chempur (Piekary Slaskie, Poland). DCM was used without further purification. DMF was distillated over ninhydrin and stored under molecular sieves 4A. HPLC solvent acetonitrile was purchased from Merck (Darmstadt, Germany). Urokinase, trypsin, kallikrein and Bzl–L–Arg–*p*NAHCl (Bzl = benzyl) were purchased from Sigma (Schnelldorf, Germany). Plasmin, S-2444 (pyro–Glu–Gly–Arg–*p*NAHCl), S-2238 (H–d-Phe–Pip–Arg–*p*NA), S-2251 (H–d-Val–Leu–Lys–*p*NA), S-2266 (H–d-Val–Leu–Arg–*p*NA2HCl and S-2288 (H–d-Ile–Pro–Arg–*p*NA) were obtained from Chromogenix (Milano, Italy). Thrombin and phosphate buffered saline (PBS) were purchased from Lubelska Wytwórnia Szczepionek (Lublin, Poland). t-PA was obtained from Boehringer Ingelheim GmbH (Ingelheim, Germany).

### Peptide Synthesis

The peptides shown in Table 1 were synthesized manually using the standard Fmoc-based strategy (Chan and White 2000). Fmoc deprotection steps were performed with 20 % (v/v) piperidine in DMF/NMP (1:1) for 3 and 8 min. separately. The peptide bonds with Fmoc amino acids were carried through urea coupling reagent TBTU in DMF/NMP/DCM (1:1:1) of amino acid/TBTU/HOBt/resin using a molar ratio 3:3:3:1. The reactions were monitored with the Steward chloranil test (Vojkovski 1995) (chlorotrityl and Rink amide resins) and with the TNBS test (1,5-diaminopentanetrityl resin) (Hancock and Battersby 1976). The cleavage from the resin was carried out with TFA/water (95/5). After 3 h stirring, the resin was filtered and washed with TFA. The combined filtrates were concentrated under reduced pressure. The crude peptide was washed with cold diethyl ether, filtered, dissolved in water and lyophilized. The Waters system (Waters Corporation, USA) was used for analytical and semipreparatory HPLC (Phenomenex C18, Jupiter 90A, 4 micron, 250 × 4 mm; Phenomenex C18, Jupiter 300A, 5 micron, 250 × 10 mm; solvents: A, 0.1 % aqueous TFA; B, 0.1 % TFA in acetonitrile, gradient 1–99 % B in A in 30 min, flow rate 1 ml/min, monitored at 220 nm). The major peak fraction was pooled and lyophilized. The molecular weight determination was performed by mass spectrometry using a Bruker Daltonics Esquire 6000 (Bruker Daltonik GmbH, Leipzig, Germany) with electrospray ionization (ESI).

**Table 1:** Physico-chemical parameters of synthesized compounds

No	Compound	Yield (%)	Retention time (min)	MW	(M + H)^+^
1	H–Phe–Lys–EACA–OH	57	18.6	406.5	407.6
2	H–D-Ala–Phe–Lys–EACA–OH	49	21.5	477.6	478.5
3	H–Ala–Phe–Lys–EACA–OH	47	21.5	477.6	478.4
4	H–D-Ala–Phe–EACA–OH	56	17.8	349.4	350.5
5	H–Ala–Phe–EACA–OH	54	17.6	349.4	350.7
6	H–Phe–Lys–EACA–NH_2_	51	19.3	405.5	406.3
7	H–D-Ala–Phe–Lys–EACA–NH_2_	48	23.6	476.6	477.4
8	H–Ala–Phe–Lys–EACA–NH_2_	50	23.9	476.6	477.8
9	H–D-Ala–Phe–EACA–NH_2_	55	18.9	348.4	349.3
10	H–Ala–Phe–EACA–NH_2_	49	18.6	348.4	349.3
11	H–Phe–Lys–EACA–NH–(CH_2_)_5_–NH_2_	42	16.9	490.7	491.2
12	H–D-Ala–Phe–Lys–EACA–NH–(CH_2_)_5_–NH_2_	38	18.5	561.8	562.4
13	H–Ala–Phe–Lys–EACA–NH–(CH_2_)_5_–NH_2_	37	18.7	561.8	562.5
14	H–D-Ala–Phe–EACA–NH–(CH_2_)_5_–NH_2_	43	16.8	433.6	434.5
15	H–Ala–Phe–EACA–NH–(CH_2_)_5_–NH_2_	41	15.9	433.6	434.1

### Enzymatic Investigations

Determination of amidolytic activity was performed as previously described by (Okada et al. 1988). Buffer and 0.1 ml of enzyme solution was added to 0.2 ml of examined compound dissolved in 0.15 M NaCl **(1**–**15**) (as control 0.15 M NaCl). The buffer and the enzyme solution included:

- tris buffer—0.6 ml (pH 8.8), enzyme: urokinase (50 units/ml), synthetic substrate: S-2444 (0.1 ml, 3 mM);
- tris buffer—0.5 ml (pH 8.4), enzyme: thrombin (1 units/ml), synthetic substrate: S-2238 (0.2 ml, 0.75 mM);
- tris buffer—0.5 ml (pH 7.4), enzyme: plasmin (0.4 units/ml), synthetic substrate: S-2251 (0.2 ml, 3 mM);
- borane buffer—0.5 ml (pH 7.5), enzyme: trypsin (0.4 units/ml), synthetic substrate: Bzl-l-Arg–pNA.HCl (0.2 ml, 8 mM);
- tris buffer—0.6 ml (pH 9.0), enzyme: kallikrein (3 units/ml), synthetic substrate: S-2266 (0.1 ml, 75 mM);
- tris buffer—0.6 ml (pH 8.4), enzyme: t-PA (167 mg/ml), synthetic substrate: S-2288 (0.1 ml, 10 mM).

The results are given in Table 2. IC_50_ values were determined (IC_50_, Inhibitor Concentration, the concentration at which enzyme activity is inhibited by 50 %). Our results were compared with the 6-aminohexanoic acid. No effect was observed in maximum concentration (20 mM) for EACA of all tested enzymes.

**Table 2:** Inhibition of synthesized tripeptides on the amidolytic activity of enzymes

No	Compound	IC_50_ (mM)				
		Plasmin	Thrombin	tPA	uPA	Trypsin
1	H–Phe-Lys–EACA–OH	11.39 ± 0.91	–	–	–	15.44 ± 1.24
2	H–D-Ala–Phe–Lys–EACA–OH	3.37 ± 0.27	3.85 ± 0.31	14.40 ± 1.15	–	–
3	H–Ala–Phe–Lys–EACA–OH	4.82 ± 0.39	5.04 ± 0.40	17.64 ± 1.41	–	–
4	H–D-Ala–Phe–EACA–OH	10.89 ± 0.87	–	6.48 ± 0.52	3.17 ± 0.25	–
5	H–Ala–Phe–EACA–OH	–	4.77 ± 0.38	–	3.38 ± 0.27	–
6	H–Phe–Lys–EACA–NH_2_	1.43 ± 0.11	–	–	–	–
7	H–D-Ala–Phe–Lys–EACA–NH_2_	0.02 ± 0.0016	–	–	–	–
8	H–Ala–Phe–Lys–EACA–NH_2_	0.13 ± 0.01	7.59 ± 0.61	–	–	–
9	H–D-Ala–Phe–EACA–NH_2_	1.55 ± 0.12	–	–	–	–
10	H–Ala–Phe–EACA–NH_2_	–	–	–	2.61 ± 0.21	–
11	H–Phe–Lys–EACA–NH–(CH_2_)_5_–NH_2_	4.82 ± 0.39	–	–	–	–
12	H–D-Ala–Phe–Lys–EACA–NH–(CH_2_)_5_–NH_2_	1.11 ± 0.09	–	–	–	–
13	H–Ala–Phe–Lys–EACA–NH–(CH_2_)_5_–NH_2_	1.4 ± 0.11	–	–	–	–
14	H–D-Ala–Phe–EACA–NH–(CH_2_)_5_–NH_2_	4.77 ± 0.38	–	–	–	–
15	H–Ala–Phe–EACA–NH–(CH_2_)_5_–NH_2_	–	–	–	–	–

(–) = No cytotoxic effect was observed in maximum concentration (20 mM). The examined compounds did not influence the enzymatic activity of kallikrein in maximum concentration (20 mM)

### Antyfibrinolytic Activity

#### Study Subject

Five healthy volunteers, men (age range: 25–34 years)) with normal blood cell counts were entered into the study. Healthy volunteers had not taken medication known to affect platelet function and/or coagulation for at least 10 days before blood sampling. Study protocol was approved by the Ethics Committee at the Medical University of Bialystok (No R-I-002/224/2015). The procedures were in accordance with the Declaration of Helsinki of 1975, as revised in 2000 and blood samples were obtained with the subjects’ informed consent.

### Blood Collection

Venous blood was collected with minimum trauma and stasis via a 21-gauge needle (0.8 × 40 mm) into 9 ml polypropylene vacuum tubes (Vacuette, Greiner Bio-One, Kremsmünster, Austria) containing 130 mM trisodium citrate. Blood was stored at room temperature for 30 min after venipuncture and then was evaluated by thromboelastometric analyses.

### Thromboelastometric (ROTEM) Analyses

**Fig. 3:**
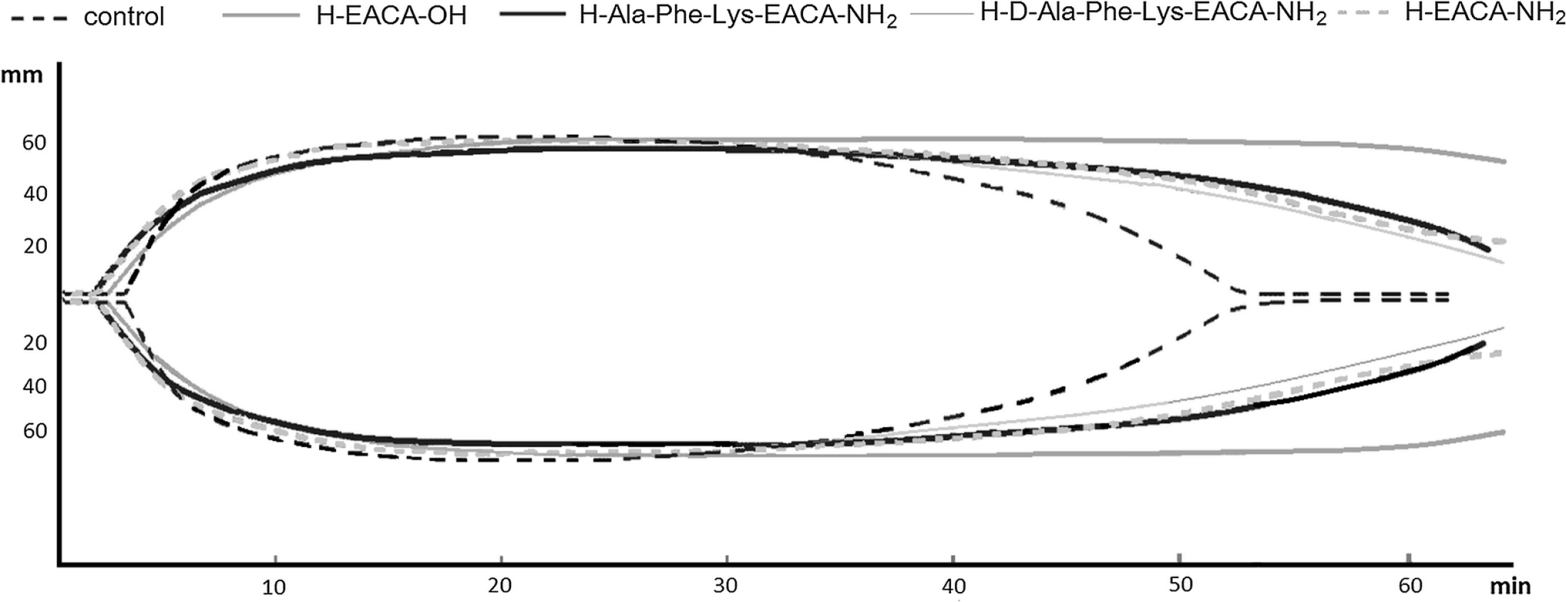
Typical thromboelastometric tracing obtained for analyzed compounds control: blood without additives. The results of one representative experiment (out of eight) are presented

ROTEM technology is described elsewhere (Luddington 2005). Thromboelastometric measurements were performed using ROTEM system (Tem International GmbH, Manheim, Germany). Recalcified (10 mM CaCl_2_) blood was assessed for fibrinolytic potential using either 140 ng/ml tissue factor (TF), and 125 ng/ml tissue PLG activator (tPA). We measured the parameters characterized kinetic of clot formation (clotting time, alpha angle), clot strength (maximal clot firmness; MCF), and fibrinolysis (percentage reduction of MCF, clot lysis time). All ROTEM measurements were performed by the same experienced operator as follows: 0.32 ml of blood (previously treated/preincubated with tested substances) was transferred into a preheated cup containing 20 μl of re-calcification reagents and repeatedly gently pipetted to mix the components (Rusak et al. 2014) (Fig. 3; Table 3).

**Table 3:** Effect of analyzed compounds on fibrinolysis parameters—thromboelastometric data

Compounds	LOT (s)	LT (min)	LI30–LI45–LI60 (%)
Control	1993 ± 111	48 ± 4	91–39–2
H–EACA–OH	≥4084	≥168	100–98–92
H–EACA–NH_2_	2488 ± 172	71 ± 5	96–80–32
H–Ala–Phe–Lys–EACA–NH_2_	2731 ± 160	76 ± 6	99–86–43
H–D-Ala–Phe–Lys–EACA–NH_2_	2519 ± 176	72 ± 4	97–79–28

*LOT* lysis onset time—time from the start of coagulation to the point which the amplitude of formed clot is reduced by 15 % (s). *LT* lysis time, when clot is reduced/lysed by 90 % (s). *LI30–LI45–LI60* Lysis index amplitude of clot (%) after 30, 45, 60 min (%). *Control* without synthesized peptides

### Antitumor Activity

#### Tissue Culture

All studies were performed on MCF-7, MDA-MB-231 and DLD cells lines were purchased from American Type Culture Collection (Rockville, MD). The cells were maintained in DMEM supplemented with 5 % fetal bovine serum (FBS), 2 mmol/ml glutamine, 50 U/ml penicillin, 50 mg/ml streptomycin at 37 °C in a 5 % CO_2_ incubator.

#### Cytotoxicity Assay

The toxicity of the evaluated peptides was determined by the method of Plumb et al. (1989) in 10, 100, 250, 500 and 1000 μM concentrations. MCF-7, MDA-MB-231 and DLD cells were maintained as described above. The detailed description of the method is given by (Markowska et al. 2013).

## Results and Discussion

For all the peptides antiamidolytic activity was evaluated against six enzymes from the group of serine proteases—plasmin, thrombin, tPA, uPA, kallikrein and trypsin. The thromboelastometric test was performed to examine the effect of the synthesised compounds on coagulation and fibrinolysis in whole blood. Additionally, the influence of peptides on the viability of fibroblast cells, colon carcinoma line DLd-1 and breast carcinoma cell lines MCF-7 and MDA-MB-231 was examined.

Out of the 15 peptides obtained, 12 inhibited the activity of plasmin with IC_50_ values ranging from 0.02 to 11.39 mM. Four compounds exhibited IC_50_ values towards thrombin ranging from 3.85 to 7.59 mM. TPA was inhibited by 3 of the synthesised compounds with IC_50_ values from 6.48 to 17.64 mM and 3 of them inhibited urokinase with IC_50_ values from 2.61 to 3.38 mM (Table. 2). The use of the characteristic –Ala–Phe–Lys– sequence of plasmin inhibition resulted in obtaining eight peptides selective for plasmin.

In the case of peptides with a free carboxyl group, peptide H–d-Ala–Phe–Lys–EACA–OH **2** showed the highest inhibition of plasmin activity with IC_50_ 3.37 mM. Peptide **2** exibited the highest activity against thrombin with IC_50_ values of 3.85 mM. It was observed that the removal of lysine from the sequence resulted in a complete loss of activity only in the case of peptide H–Ala–Phe–EACA–OH **5** containing l-alanine whereas in the case of peptide 4 with alanine in the D configuration, the activity was merely reduced with the IC_50_ value of 10.89 mM.

The highest inhibition of plasmin activity was exhibited by the amide of peptide H–d-Ala–Phe–Lys–EACA–NH_2_ **7**, which also proved to be the most active compound of all the synthesised compounds of the **1–15** series. Peptides H–Phe–Lys–EACA–NH_2_ **6**, H–Ala–Phe–Lys–EACA–NH_2_ **8** and H–d-Ala–Phe–EACA–NH_2_ **9** inhibited plasmin activity with IC_50_ values of 1.43 mM, 0.13 mM, and 1.55 mM respectively. It was also observed that in this group the removal of lysine from the sequence resulted in a complete loss of activity only in the case of peptide **10** containing l-alanine.

Another series of compounds were peptides with secondary amides with the 1,5-diaminopentyl moieties (fragment of the natural diamine—cadaverine). Midura-Nowaczek et al. 1990 proposed the insertion of plasmin 1,5-diaminopentyl residue as a form of decarboxylated lysine—cadaverine into the structures of peptide inhibitors. Use of this fragment was based on thrombin inhibitors containing the decarboxylated structure—agmatine in the place of the C*-*terminal arginine (Rewinkel and Adang 1999). A previously synthesised amide derivative of lysine (Ac–Lys–NH–(CH_2_)_5_–NH_2_) inhibited plasmin activity with IC_50_ values of 8 mM (Midura-Nowaczek et al. 2008). In contrast, a peptide with a similar sequence to the analysed H–Ala–Phe–Lys–NH–(CH_2_)_5_–NH_2_ showed no plasmin inhibitory activity (Midura-Nowaczek et al. 1990). The most active inhibitor of plasmin activity in the described cadaverine series was peptide H–d-Ala–Phe–Lys–EACA–NH–(CH_2_)_5_NH_2_ **12** with IC_50_ 1.11 mM. The removal of lysine from the sequence resulted in H–Ala–Phe–EACA–NH–(CH_2_)_5_NH_2_ **15** not inhibiting plasmin.

We describe five different peptide sequences in this paper. In our tests the inhibitors containing the *N-*terminal d-Ala proved marginally more active than those containing l-Ala. IC_50_ values were in the range of 0.02 mM **7** to 3.37 mM **2** for peptides with d-Ala and from 0.13 mM **8** to 4.82 mM **3** in the case of l-Ala.

We also compared the activity of some of the peptides synthesised with the literature data regarding similar peptides containing EACA (–Ala–Phe–Lys– vs. –Ala–Phe–Lys–EACA–). Compounds with a free carboxyl group of H–d-Ala–Phe–Lys–EACA–OH **2** and H–Ala–Phe–Lys–EACA–OH **3** exerted an enhanced plasmin inhibitory activity effect compared to the corresponding H–d-Ala–Phe–Lys–OH and H–Ala–Phe–Lys–OH. The IC_50_ values of the analysed compounds containing d-alanine or l-alanine were lower in comparison with the values of the compounds previously synthesised by Markowska et al. (2007).

Replacement of the lysine on EACA in parent sequence (–Ala–Phe–Lys– vs. –Ala–Phe–EACA–) resulted in growth inhibition towards plasmin. In the case of amides of peptides H–d-Ala–Phe–EACA–NH_2_ **9** and H–d-Ala–Phe–EACA–NH–(CH_2_)_5_–NH_2_ **14** a significant increase of inhibition was observed (IC_50_ = 1.55 mM for **9**, IC_50_ = 4.77 mM for **14**, no activity for H–d-Ala–Phe–Lys–NH_2_ (Markowska et al. (2007)) and for H–d-Ala–Phe–Lys–NH–(CH_2_)_5_–NH_2_ (Midura-Nowaczek et al. 1990).

Amides of the synthesised peptide H–d-Ala–Phe–Lys–EACA–NH_2_ **7** and H–Ala–Phe–Lys–EACA–NH_2_ **8** exhibited a significant inhibitory effect on plasmin activity (IC_50_ = 0.02 mM and IC_50_ = 0.13 mM) while compounds with *C*-terminal lysine H–d-Ala–Phe–Lys–NH_2_ and H–Ala–Phe–Lys–NH_2_ completely lacked this effect (Midura-Nowaczek et al. 2003).

In the case of cadverine derivatives of the synthesised compounds it was possible to compare only the compound H–Phe–Lys–EACA–NH–(CH_2_)_5_–NH_2_ **11** with unpublished data regarding the compound H–Phe–Lys–NH–(CH_2_)_5_–NH_2_ available at the Department of Organic Chemistry of Bialystok Medical University. Peptide **11** with IC_50_ = 4.82 mM proved significantly more active than the corresponding compound without EACA.

Due to the antifibrinolytic effect of ε-aminocaproic acid, the tromboelastometric test was conducted to investigate the effect exerted by the peptides on the kinetics of clot formation and stability in human blood.

The selected compounds did not influence coagulation parameters in the thromboelastometric assay. Only in the case of H–d-Ala–Phe–Lys–EACA–NH_2_ **7** 115 s (1.9 min.) and H–Ala–Phe–Lys–EACA–NH_2_ **8** 135 s (2.25 min.), a marginal reduction in the control clotting time (CT—coagulation time) 174 s (2.9 min.) was observed. EACA and its amide demonstrated a similar effect (137 and 117 s respectively). It did not have any impact on the activation of prothrombin exhibited by the absence of a significant change in the α angle (68–73° to control 75°).

In all cases a clot was formed with similar consistency as evidenced by the unchanged value of maximal clot firmness (MCF) (56–59 relative to control 60). It was observed that in the presence of the synthesised compounds fibrinolysis of the formed clot occurred more slowly (LI—lyse index, expressed in  %). Comparing the value of lysis in 45 min (LI_45_) the whole blood lysis is about 61 % of the clot. In the presence of H–EACA–OH fibrinolysis was almost completely inhibited (1 %) while in the presence of H–EACA–NH_2_ (20 %), H–Ala–Phe–Lys–EACA–NH_2_ **8** (14 %) and the H–d-Ala–Phe–Lys–EACA–NH_2_ **7** (21 %) it was reduced.

The time at which the clot decreased by 15 % (parameter LOT—lysis onset time) and 90 % (parameter LT—lysis time) was also examined. In the case of the synthesised compounds an increase in LOT relative to the control was observed. The clot decreased by 15 % after approximately 45 min (2731 s) and 90 % after about 67 min (4060 s) in the case of H–Ala–Phe–Lys–EACA–NH_2_ **8**, while in the presence of tPA in pure blood fibrinolysis was at the level of 15 % after approximately 33 min (1993 s), and 90 % after about 48 min (2912 s). A similar effect was observed for the EACA amide and H–d-Ala–Phe–Lys–EACA–NH_2_ **7**. In the case of EACA these parameters were not determined due to its antifibrinolytic activity.

In conclusion, the synthesised peptides produced a less marked antifibrinolytic effect compared with EACA, but the effect was more profound in comparison with the control. The most probable reason for a reduction in this activity is the inhibition of peptide interaction with LBS of plasmin/PLG achieved by blocking the amino group in EACA through amide bond with tripeptide –Ala–Phe–Lys– and blocking the carboxyl group as an amide.

Owing to the established anti-tumor properties of the –Ala–Phe–Lys– sequence present in the synthesised peptides, impact of peptides on healthy cells—fibroblasts, and cancerous cells—colon carcinoma cell lines DLD-1 breast cancer cells MCF-7 and MDA-MB -231 was also examined.

The peptide –Ala–Phe–Lys– was inserted into the structure of prodrugs recognised by plasmin to selectively strengthen their anti-tumor activity and to reduce side effects of the potential therapeutic agent. Plasmin, whose large quantities occur in cancer cells (and other components of the PLG activation system), recognises the sequence –Ala–Phe–Lys and releases the active drug through a hydrolysis reaction. The prodrugs of doxorubicin(Takemura and Fujiwara 2007), camptothecin (De Groot et al. 2002), paclitaxel (De Groot et al. 2000), daunorubicin (De Groot et al. 1999) and methotrexate (Warnecke et al. 2007) proposed by various authors have displayed increased cytotoxicity against cancer cell line MCF-7, EVSA-T, WIDR IGROV, M19, A498, and H226 in cell assays.

The described peptides did not exert any cytotoxic effects on normal cells and cancer cells. Referring to the mechanism of action of de Groot’s prodrugs it is not possible to observe bond hydrolysis in the described inhibitors between EACA and lysine due to the fact that plasmin is an endopeptidase and does not hydrolyse the *C*-terminal amino acids of peptides or proteins (McDonald 1985). In fact, in contrast to the hydrolysis of synthetic substrates used in the enzymatic experiments, mass spectrometry confirmed that there was no hydrolysis of EACA of the peptides fragments. Therefore, it is impossible to suggest a mechanism of action of the described inhibitors similar to that of de Groot’s anticancer prodrugs. The mechanism of action of peptides presented in the paper is based on direct interaction with the active site and “lysine binding sites” in plasmin. Nevertheless, the presence of C-terminal EACA may be responsible for reduced cytotoxicity to the tested cells.

The reported findings are the result of basic research and require further testing.

## Conclusion

 Peptide derivatives of plasmin inhibitors containing the EACA and (-Ala–Phe–Lys-) with biological antiamidolytic and antifibrynolytic activity were synthesised. The peptide H–d−Ala–Phe–Lys–EACA–NH_2_ **8** was found to be a selective inhibitor of plasmin compared to other examined serine proteases. The highest fibrinolytic activity was displayed by H–d-Ala–Phe–Lys–EACA–NH_2_ **8** and H–Ala–Phe–Lys–EACA–NH_2_ **7**. Nevertheless, it was less profound than the fibrinolytic activity of EACA, which may be due to dimension misalignment and lack of interactions between the anionic and cationic centres in the lysine-binding sites. Despite the presence of the sequence -Ala–Phe–Lys accepted to be responsible for the enhanced antitumor properties of known prodrugs, no cytotoxicity against either fibroblasts or cancer cell line (colon carcinoma line DLD-1 and breast carcinoma lines MCF-7 and MDA-MB-231) was observed. This may suggest that the presence of C-terminal EACA, which did not display cytotoxicity in our tests, in the synthesised peptides may be responsible for increasing the viability of the tested cells.
